# Identifying a glucose metabolic brain pattern in an adeno-associated viral vector based rat model for Parkinson’s disease using ^18^F-FDG PET imaging

**DOI:** 10.1038/s41598-019-48713-0

**Published:** 2019-08-26

**Authors:** Martijn Devrome, Cindy Casteels, Anke Van der Perren, Koen Van Laere, Veerle Baekelandt, Michel Koole

**Affiliations:** 1 Department of Nuclear Medicine and Molecular Imaging, KU Leuven, Leuven, Belgium; 2 Laboratory for Neurobiology and Gene Therapy, Department of Neurosciences, KU Leuven, Leuven, Belgium

**Keywords:** Parkinson's disease, Preclinical research

## Abstract

We investigated the glucose metabolism in an adeno-associated viral vector based alpha-synuclein rat model for Parkinson’s disease (PD) using longitudinal ^18^F-FDG PET imaging, which resulted in an improved characterization of this animal model. We generated a PD specific pattern (PDSP) based on a multivariate classification approach to differentiate between a PD and control group at a late disease stage, where the neurodegeneration is considered nearly complete. In particular, we applied a principal component analysis prior to classification by a support vector machine (SVM). Moreover, by using a SVM for regression to predict corresponding motor scores, a PD motor pattern (PDMP) was derived as well. The PDSP mainly corresponds to the PDMP and overlaps to a large extent with the human pattern. We were able to quantify disease expression at previous time points by projecting onto the PDSP and PDMP. While a univariate analysis indicated metabolic changes which did not persist through time, both PDSP and PDMP were able to differentiate significantly (p-value < 0.05) between the PD and control group at week 4, 6 and 9 post injection, while no significant differences were obtained at baseline and at week 3, which is in accordance with the animal model.

## Introduction

Parkinson’s disease (PD) is a progressive neurodegenerative movement disorder that typically occurs in elderly people and affects about one percent of persons older than 60 years^1^. Clinical motor symptoms are characterized by tremor, bradykinesia, and muscle rigidity along with impaired gait and posture, while other clinical manifestations also observed at the time of diagnosis and referred to as the non-motor symptoms, include autonomic dysfunction, cognitive disorders, and sensory and sleep abnormalities^2^. The pathological hallmark of PD is represented by progressive dopaminergic neuronal loss in the Substantia Nigra pars compacta (SNpc), resulting in striatal depletion of dopamine transmission^3^. Although the exact mechanism of dopaminergic (DA) neuronal loss in the SN is not well understood, protein misfolding of alpha-synuclein and subsequent intracellular accumulation has become a leading hypothesis^4,5^.

In order to identify preclinical nigrostriatal dysfunction and to quantify DA terminal functionality to determine the stage of DA degeneration, PET imaging of the DA pathway can be considered^6^. However, the motor and cognitive symptoms of PD cannot be attributed merely to striatal dopaminergic dysfunction. Therefore, functional imaging approaches such as ^18^F-FDG PET are considered to reveal functional abnormalities in neuronal circuits such as the cortico-striato-pallido-thalamocortical (CSPTC) loops and related pathways^7,8^ which could be affected by presynaptic nigrostriatal DA cell loss^9,10^. Based on ^18^F-FDG brain PET imaging, a human Parkinson’s disease-related pattern (PDRP), representing PD specific brain regions of hyper- and hypometabolism, was derived by using the Scaled Subprofile Model (SSM)^11^ which is based on a principal component analysis prior to classification by logistic regression between PD patients and healthy controls^12^. Besides the PDSP, other specific PDRPs were identified, such as the Parkinson disease Tremor-Related Pattern (PDTP)^13^ and the Cognition-Related Pattern (PDCP)^14,15^. Hence, ^18^F-FDG brain PET imaging provides PD related metabolic signatures, or imaging biomarkers, representing brain regions which become less or more metabolic active as a consequence of dopaminergic neurodegeneration in the SN^16,17^.

The aim of this study was to use longitudinal small animal ^18^F-FDG PET imaging to determine a PD specific glucose metabolic brain pattern for a PD rat model. Today, no preclinical PD specific glucose metabolic brain pattern has been identified, only aberrant regional metabolism in PD-associated brain regions has been described in acute models^18^. PD rat models that closely resemble the neuropathology, physiology and motor symptoms of human PD are essential to further investigate the molecular pathophysiology of PD and to develop novel therapeutic strategies. For this study, we focused on a rat model for Parkinson’s disease based on overexpression of alpha-synuclein with adeno-associated viral vectors^19^. This rat model is based on a direct stereotactic injection of adeno-associated viral vectors serotype 2/7 (rAAV2/7) encoding for the human A53T α-synuclein (α-SYN) in the substantia nigra (SN). This resulted in a progressive PD animal model with reproducible nigrostriatal pathology and behavioral deficits within four weeks and nearly complete dopaminergic cell loss within an acceptable time frame of five weeks, while L-DOPA treatment was found to reverse the behavioral phenotype. The validity of the α-SYN rAAV2/7 PD rat model has been demonstrated by extensive histopathological and biochemical analysis, motor behavior testing and *in vivo* microdialysis, including non-invasive longitudinal monitoring of neurodegeneration using PET imaging of the dopamine transporter (DAT) and magnetic resonance spectroscopy (MRS)^19^.

A univariate analysis was applied on the same longitudinal PD rat model to investigate the glucose metabolism with ^18^F-FDG PET imaging. However, the observed metabolic changes did not persist through time at late disease stages as would be expected in degenerative diseases. Therefore, we considered a machine learning approach, which is fundamentally different from mass univariate analysis techniques. More specifically, when the PD rat model was at a late disease state, ^18^F-FDG PET and motor behavioral data were used to generate a glucose metabolic brain pattern either by classification between the PD and control group (discriminative PD specific pattern) or by applying machine learning regression to predict the motor scores (motor-related PD pattern). The metabolic brain patterns were compared with human PDRPs of glucose hyper- and hypometabolism. Additionally, pattern expression scores were determined at different stages of the PD model to evaluate whether the scores are in line with disease progression and to identify at which stage of the PD rat model disease expression is manifested metabolically.

## Methods

### α-SYN rAAV2/7 PD rat model

All experiments were conducted on 18 female Wistar rats (body weight ranged from 204 to 243 g at the start of the study). We considered a similar data set in terms of size and sex as described by Van der Perren *et al*. who characterized the rAAV2/7 PD rat model in order not to introduce extra variability in the anima model^19^. Ten rats were stereotactically injected with rAAV2/7 encoding for the human A53T α-synuclein mutant in the right SN (PD group), while the other 8 were stereotactically injected with rAAV2/7 encoding enhanced green fluorescent protein (eGFP, control group). All animals were injected with 3 µl (9.0 E11 genome copies/ml) rAAV vector. Stereotactic coordinates used for the SN of the rat were anteroposterior (AP) −5.3, lateral (LAT) −2.0, dorsoventral (DV) −7.2 calculated from the dura using bregma as reference. All rats were housed per 2 or 3 per genotype, at an average room temperature of 22 °C and a 12/12-h light/dark cycle. Food and water were given ad libitum. The research protocol was approved by the local Animal Ethics Committee of the University of Leuven and was according to European Ethics Committee guidelines (decree 86/609/EEC). PET scans and behavioral tests were repeated at baseline before stereotactic injection and at 3, 4, 6 and 9 weeks after rAAV2/7 injection.

### ^18^F-FDG PET data acquisition and preprocessing

Cerebral FDG images were obtained for all 18 animals using ^18^F-FDG, prepared by a standard synthesis module (IBA, Louvain-la-Neuve, Belgium). Small animal PET imaging was performed using a lutetium oxyorthosilicate detector-based FOCUS 220 tomograph (Siemens/Concorde Microsystems, Knoxville, TN, USA), which has a transaxial resolution of 1.35 mm full-width at half-maximum (FWHM). Data were acquired in list mode in a 128 × 128 × 95 matrix with a pixel width of 0.475 mm and a slice thickness of 0.796 mm. The coincidence window width was set at 6 ns. The rats were anesthetized with 2% isoflurane in 2.0 L/min oxygen before being injected with the radioligand. Tail veins were catheterized to enable the infusion of ^18^F-FDG (22 ± 3 MBq; specific activity range 83–710 GBq/μmol). The radioligand was diluted with saline to obtain a 5% ethanol solution and injected in a total volume of approximately 500 μl. Acquisitions were performed for 30 min, starting 60 min post injection, as previously described^20^. For quantification purposes, PET scans were reconstructed using an iterative maximum a posteriori probability algorithm with ordered subsets (MAP; 18 iterations, 9 subsets, fixed resolution: 1.5 mm). PET images were spatially normalized for each animal onto the Schiffer rat brain atlas (version 3.9, PMOD Inc., Zurich, Switzerland). After smoothing with a Gaussian isotropic kernel with FWHM of 1.6 mm and masking, the images were vectorized, a demean (subtracting the mean uptake) was performed and finally normalized by dividing by the *L*_2_ norm (sum of squares equals one).

### Motor behavior scores using the cylinder test

The cylinder test, considered as the gold standard to detect motor deficits in unilateral PD models, was performed at each time point on both alpha-synuclein and control rats to assess the asymmetry of forelimb use during explorative activity. The rats were placed in a transparent glass cylinder (40 cm height and 20 cm diameter) and videotaped while they explored the cylinder walls with their forelimbs. The number of forelimb contacts with the cylinder wall were counted until a minimum of 20 contacts was recorded. Contact was determined by placement of either the right or left forepaw. Data were expressed as percent use of the impaired forelimb relative to the total number of wall contacts, and given in the supplementary material.

### Glucose metabolic brain patterns for the α-SYN rAAV2/7 PD rat model

Both the PD specific pattern (PDSP) and PD motor pattern (PDMP) were generated based on a principal component analysis (PCA) prior to classification or regression by a support vector machine (SVM) with linear kernel. The general framework for both approaches is given by the generalized linear model (GLM)^21^. Consider a vectorized *D*--dimensional medical image *x* with corresponding discrete class label or continuous score *t* for classification and regression respectively. In case of regression, for a GLM combined with a linear feature transform characterized by the transformation matrix *A*, the estimated continuous output score *y* is given by

1 $$y(x)={w}^{T}Ax+b,$$

where the weight vector *w* and the bias parameter *b* are determined by a training data set of size *N*: {*x*_*n*_, *t*_*n*_} (*n* = 1, …, *N*). In case of (deterministic) binary classification, the step function acts upon the argument *w*^*T*^ *Ax* + b, such that the output variable *y* is a binary value. Rewriting the above Eq. (1), gives the following expression:

2 $$y(x)=\langle {A}^{T}w,\,x\rangle +b,$$

demonstrating that the output *y* is estimated upon taking the inner product between *A*^*T*^*w* and the subject *x*. Therefore, in case a proper classification/prediction model and feature transform is applied, the vector *A*^*T*^*w* could be interpreted as a PD discriminative brain pattern (imaging biomarker) if a classification is performed between a PD and control group and a PD motor related brain pattern in case motor scores are estimated. Moreover, projecting the scan *x* onto the vector *A*^*T*^*w* by taking the inner product *A*^*T*^*w*, *x* contains a measure of pattern expression. The weight vector ***w*** and bias *b* are obtained by a loss function penalizing the error made by modeling the real output value *t* by the estimated value *y*, in combination with a regularization function to avoid overfitting.

For this study, we opted for a support vector machine (SVM) model with linear kernel and *L*_1_-norm soft margin for classification^22^, and SVM with *L*_1_ loss for regression^23^. A PCA was performed as feature transform on the full training data set including both the PD and control groups prior to classification/prediction by SVM, generating an orthonormal space spanned by all principal components. As such, the rows of the transformation matrix *A* are given by the principal components, determined by the eigenvectors of the data covariance matrix^24,25^. Consequently, the pattern *A*^*T*^*w* is generated by a linear combination of these principal components. The SVM minimization problem for both the binary classification and regression, being a quadratic programming problem in dual space, was solved by sequential minimal optimization^26^ (MATLAB version 2016b, The MathWorks Inc., Massachussetts, United States). The kernel scale and soft margin hyperparameters were optimized using a grid search and a nested inner loop 10 fold cross-validation.

To generate a metabolic PDSP and PDMP, both classification and regression were performed using the PET data and motor behavior scores of week 9, corresponding to near-complete and stable dopaminergic degeneration. The accuracy of the binary classifier separating the PD group from controls at week 9 was evaluated by leave-one-out cross-validation. Pattern expression scores at earlier time points were determined by taking the inner product with the patterns derived at week 9, allowing to metabolically quantify disease expression. Group differences between the PD and control group (at baseline, week 3, week 4, week 6 and week 9) in terms of pattern expression scores were evaluated by a Wilcoxon rank sum test (5% significance level).

Since rats from the PD and control group match a positive and negative pattern score respectively, regions in the PDSP with a predominant positive weighting correspond to an increased glucose metabolism relative to healthy rats, whereas a predominant negative weighting represent glucose hypometabolic regions.

## Results

**Figure 1:**
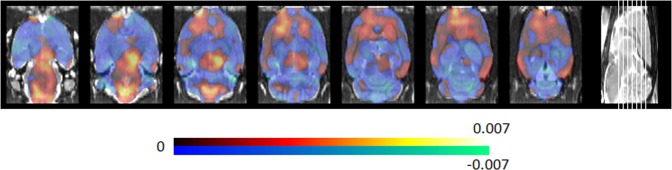
Metabolic PDSP derived by classification between PD group and controls for the α-SYN rAAV2/7 PD rat model. The PDSP indicates discriminative regions of relative hypermetabolism (positive weights indicated in yellow/red) and hypometabolism (negative weights indicated in blue). The PDSP is based on the ^18^F-FDG-PET scans at week 9, where the dopaminergic degeneration is considered stable and near-complete.

**Figure 2:**
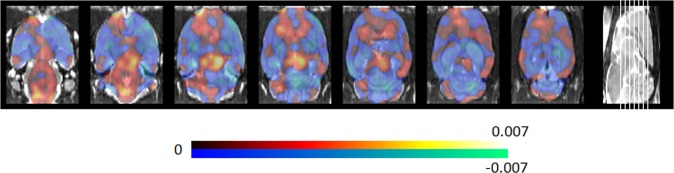
Metabolic PDMP obtained by SVM regression of the behavioral motor scores of both the PD and control group for the α-SYN rAAV2/7 PD rat model. Positive weights (red/yellow) contribute to a high motor score (cylinder test) and therefore indicating regions of hypermetabolism, whereas negative weights (blue) contribute to a lower score, indicating hypometabolic regions. The PDMP is obtained with the ^18^F-FDG-PET scans at week 9, where the dopaminergic degeneration is considered quasi-stable and near-complete.

The PD specific metabolic brain pattern determined by using all data at week 9 is given in Fig. 1, whereas the percentage of positive and negative weighting relative to the whole PDSP is summarized in Table 1 for all relevant brain regions (Schiffer atlas^27^). Regions with a predominant positive weighting relative to the PDSP, and therefore associated increased glucose metabolism, include the bilateral striatum, bilateral cortices (including motor-, somatosensory-, cingulate-, orbitofrontal-, visual-, and prefrontal cortex), midbrain, pons and medulla. Regions with predominant negative relative weighting, and thus associated decreased glucose metabolism, were noticed in thalamic, hippocampal and cerebellar regions, as well as entorhinal and insular cortices. The PD motoric brain pattern is illustrated in Fig. 2, with the corresponding percentage of positive and negative weighting relative to the PDMP summarized in Table 1. The main discrepancy with the PDSP is that the motor, orbitofrontal, somatosensory and visual cortex, striatum and thalamus are no longer predominant. However, all predominant regions in the PDMP correspond with the PDSP. Pearson correlation for voxel values within brain regions between PDSP and PDMP are given in Table 2. Since PDSP and PDMP pattern expression scores are obtained by projecting onto the PDSP and PDMP respectively and the patterns are highly correlated, PDSP and PDMP expression scores PD and control rats are correlated as well (correlation between all scores of PD and control rats combined). Pearson correlation values between PDSP and PDMP expression scores are 0.70 (baseline), 0.86 (week 3), 0.75 (week 4), 0.75 (week 6) and 0.87 (week 9).

**Table 1:** Percentage of positive and negative weighting relative to the whole brain pattern (given in Fig. 1) for different brain regions for both the PDSP and PDMP. Weighting values are used to determine whether weighting is predominantly positive or negative and therefore can be interpreted as hyper- or hypometabolic.

	PDSP			PDMP		
	Positive weighting (%)	Negative weighting (%)	Predominant	Positive weighting (%)	Negative weighting (%)	Predominant
Cingulate Cortex	3.8	0.10	+	2.3	0.095	+
Entorhinal Cortex	1.4	7.7	−	2.3	5.8	−
Insular Cortex	0.51	3.0	−	0.15	5.2	−
Medial Prefrontal Cortex	1.9	0.03	+	0.81	0.22	+
Motor Cortex	4.1	1.1	+	1.8	2.7	/
Orbitofrontal Cortex	3.4	0.84	+	2.2	1.9	/
Somatosensory Cortex	4.1	3.0	/	4.2	4.3	/
Visual Cortex	5.5	1.4	+	3.9	2.8	/
Cerebellum	1.7	3.7	−	1.9	4.4	−
Striatum	8.5	2.1	+	6.2	4.9	/
Thalamus	1.1	3.4	−	2.4	3.3	/
Hippocampus	0.055	2.2	−	0.046	1.8	−
Midbrain	4.1	0.21	+	5.2	0.10	+
Pons	3.1	0.24	+	2.6	0.14	+
Medulla	7.8	0.078	+	8.1	0.07	+

**Table 2:** Pearson correlation for voxel values within brain regions between PDSP and PDMP.

Cingulate Cortex	0.64
Entorhinal Cortex	0.72
Insular Cortex	0.86
Medial Prefrontal Cortex	0.87
Motor Cortex	0.66
Orbitofrontal Cortex	0.87
Somatosensory Cortex	0.66
Visual Cortex	0.87
Cerebellum	0.71
Striatum	0.92
Thalamus	0.78
Hippocampus	0.84
Midbrain	0.87
Pons	0.79
Medulla	0.88

**Figure 3:**
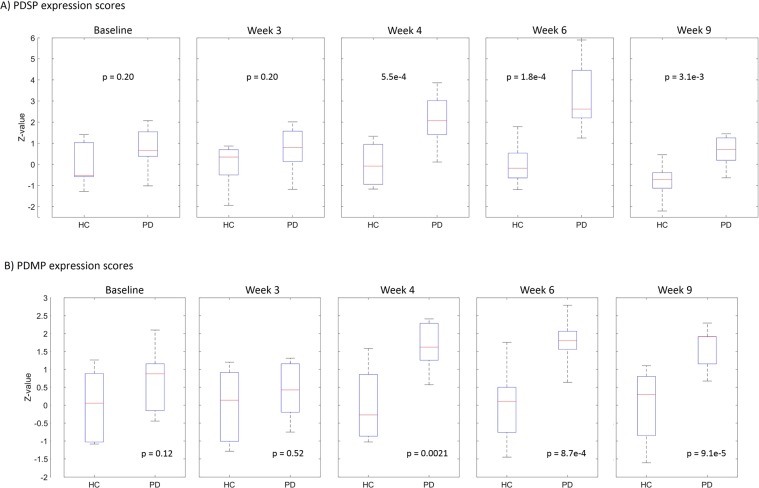
PDSP and PDMP expression scores for PD and control group at baseline, week3, week 4, week 6 and week 9. Scores (Z-values) are determined by projecting onto the PDSP (**A**) and PDMP (**B**) derived at week 9. At week 9, leave-one-out cross-validation is performed to calculate the expression scores. On each box, the central mark indicates the median, and the bottom and top edges of the box indicate the 25th and 75th percentiles, respectively, while the whiskers extend to the most extreme data points. P-values of the Wilcoxon rank sum test between the PD and control group (HC) for the PDSP and PDMP expression scores at each time point are also given.

Binary classification at week 9 between the PD group and controls resulted in a prediction accuracy of 90% (9/10) and 75% (6/8) for the PD and control group respectively. The corresponding PDSP expression scores (Z-values) obtained by projecting the PET data at week 9 onto the PDSP (Fig. 1), using leave-one-out cross-validation, are given in Fig. 3A. Z-values are calculated based on the same mean and standard deviation of all control scans (across all time points). Moreover, the pattern expression scores at baseline and earlier disease stages (week 3, 4 and 6) are also shown in Fig. 3A. The Wilcoxon rank sum test (5% significance level, p-values indicated in Fig. 3A) revealed significant differences between the PD and control group at week 4, week 6 and week 9 (p-value of 5.5e-4, 1.8e-4 and 3.1e-3 respectively) while no significant difference was observed between the PD and control group at week 3 and baseline (both p-value of 0.20), in line with the pathophysiology of this animal model. Besides projecting the PET data onto the PDSP, PD motor pattern expression scores are also calculated. Z-values of the PDMP expression scores are illustrated in Fig. 3B and corresponding p-values for the Wilcoxon rank sum test are given in Fig. 3B. The p-values for baseline (0.12) and week 3 (0.52) are not significant, while for week 4 (0.0021), week 6 (8.7e-4) and week 9 (9.1e-5), a significant difference is found between PD and control groups, in correspondence with the PDSP expression scores.

## Discussion

α-synuclein is considered a key player in the pathogenesis of PD since it is the main protein component of Lewy bodies, a pathological hallmark of PD. In some familial cases of PD this can be explained either gene duplication or triplication, or to a point mutation (A53T, A30P, E46K, H50Q or G51D) presumably leading to an abnormally folded form of α-synuclein^28–30^. This process is mimicked by stereotactic injection of rAAV2/7 encoding for the human A53T α-SYN into the rat or mouse SN^31^. Previous studies have shown that this induces overexpression of α-SYN leading to a progressive degeneration of the nigral dopaminergic neurons, along with the development of motor impairments^19^. In this study, we investigated the brain glucose metabolism using ^18^F-FDG PET in a PD rat model by using a multivariate approach. More specifically, a PCA was applied prior to classification/regression analysis by SVM to evaluate alterations in the brain glucose metabolism in the α-SYN rAAV2/7 PD rat model relative to control animals. While SVM is a widely used machine learning method in neuroimaging, PCA has proven its usefulness for PET imaging^32^ and is beneficial for reducing the impact of noise on classification or regression performance and improving the interpretability of disease-related glucose metabolic brain patterns (i.e. linear combination of principal components). The SSM has often been applied to identify metabolic disease-specific patterns and corresponding network expression scores, such as to generate a Parkinson’s disease-related pattern based on ^18^F-FDG PET data^32^. However, the SSM/PCA approach includes several preprocessing steps other than a PCA, such as double demean and log transform, and selects the relevant principal components for the disease-specific glucose metabolic brain pattern prior to classification by logistic regression^12^. On the other hand, our approach automatically assigned a weight for every principle component during the training step as expressed by Eq. (2). Moreover, a multivariate analysis is fundamentally different from a mass univariate approach where voxelwise statistical testing is used to identify clusters which are significantly different between groups. Instead, we used a machine learning approach to generate a glucose metabolic brain pattern by training a classification or prediction model. In this way, a PD specific metabolic pattern was generated by performing a classification between the PD and control animals for the for the α-SYN rAAV2/7 PD rat model. Similarly, a PD motoric pattern was generated by applying a machine learning regression of behavioral motor parameters (cylinder test scores). Since PD and control animals correspond to a positive and negative pattern expression score respectively, positive weights in these brain patterns constitute PD related hypermetabolism while negative pattern weights correspond to PD-induced hypometabolic brain regions. As such, the PDSP represented hypermetabolism in all cortical regions, except the entorhinal, insular and ipsilateral somatosensory cortex, together with hypermetabolism in striatum, midbrain, pons and medulla while hypometabolism was observed in thalamus, hippocampus and cerebellum (Table 1). Moreover, longitudinal PDSP expression scores were significantly different between the PD and control group at time points which are in line with the pathophysiology of this animal model (week 4, week 6 and week 9, see Fig. 3). Furthermore, these findings imply that the PDSP reflects PD related pathogenesis. For the PDMP, hypermetabolism was observed in the cingulate and medial prefrontal cortex, midbrain, pons and medulla, whereas hypometabolism was mainly identified in the cerebellum and hippocampus (Table 1). Most hyper- and hypometabolic brain regions of the PDSP and PDMP overlap as illustrated by the high correlation values for region-weights given in Table 2. However, for the PDMP more regions are less clearly hyper- or hypometabolic since more regions have both a large contribution of positive and negative weighting values (e.g. striatum, see Table 1). While the multivariate PDSP was able to differentiate between the PD and control group at time points in accordance with the animal model, the univariate analysis did not persist through time at late disease stages as would be expected in degenerative diseases^33^. More specifically, voxel-based analysis of relative ^18^F-FDG uptake showed a dynamic pattern of PD-related metabolic changes. At week 4, hypermetabolism is found in a cluster covering the ipsilateral nigra-thalamic region, whereas hypometabolism was noted in the ipsilateral striatum at week 6. Elevated ^18^F-FDG uptake was seen in a cluster extending across the contralateral striatum, motor- and somatosensory cortex at week 9 (Statisitcal Parametric Mapping analysis, T-maps interrogated at a p_height_ ≤ 0.005 peak level and extend threshold of k_E_ > 200 voxels; only significant clusters with p_height_ < 0.05 corrected for multiple comparisons were retained^34^).

The spatial topography of the glucose metabolic PDSP and PDMP conforms well to changes in regional synaptic activity described in other animal models of PD. In nonhuman primate MPTP models, abnormal metabolic activity was also observed with putamen/pallidum, pons and sensorimotor cortex demonstrating increased metabolic activity, while the posterior-occipital regions had a decreased metabolic activity^35^. However, the thalamic hypermetabolism which was observed in nonhuman primate MPTP models, was not present in the PDSP and PDMP of α-SYN rAAV2/7 PD rat model. On the other hand, the cerebellar hypometabolism in the brain patterns for α-SYN rAAV2/7 PD rat model is the major discrepancy with human PDRP glucose metabolic brain pattern characterized by an increased metabolic activity in the putamen, thalamus, pons, cerebellum, primary motor and sensorimotor cortex and reduced glucose metabolism in the lateral premotor cortex and parieto-occipital association regions^36^. Especially, an increased cerebellar glucose metabolism is a common physiopathological feature of PD, as shown by several ^18^F-FDG PET studies^37^. This has been interpreted as compensatory on the dysfunctional basal ganglia loop system in PD and confirmed by the normalization of cerebellar hypermetabolism after deep brain stimulation of the subthalamic nucleus^38,39^. On the other hand, a PD related cognitive glucose metabolic brain pattern, characterized by the group of Eidelberg^14^, included severe increase of cerebellar glucose metabolism. As such, it could be hypothesized that the effects of injecting rAAV2/7 encoding for α-SYN have limited effect on the cognitive function of rats. However, this discrepancy can arise from a combination of factors such as species variation, disease models, the use of anesthetics, imaging acquisition and reconstruction, post-processing and analytical methodology. Moreover, we should emphasize the difference between patterns of transient degeneration, as induced in the α-SYN rAAV2/7 PD rat model, and patterns resulting from true disease-based progressive degeneration. The same effects presumably play a role in the discrepancy between the increased thalamic glucose metabolism in human PDRP and lower glucose metabolism for the α-SYN rAAV2/7 PD rat model. A large number of cognitive tests^40^ such as attention deficit tests^41^ exist to examine the cognitive scores of the α-SYN rAAV2/7 PD rats and to evaluate to which extent motor or cognitive impairment underlie the cerebellar glucose metabolic function in the α-SYN rAAV2/7 PD rat model. On the other hand, an increase in hippocampal activity is seen in advanced human PD^42^ while the injection of rAAV2/7 encoding for α-SYN in the SN could also deplete dopaminergic neurons of the ventral tegmental area projecting to the hippocampus. This could explain the loss of hippocampal glucose metabolic function compared to the human PDRP glucose metabolic brain pattern.

## Conclusion

Based on longitudinal ^18^F-FDG PET imaging, a multivariate approach was able to generate PD specific and PD motor patterns, which resulted in an improved characterization of the glucose metabolism for the α-SYN rAAV2/7 PD small animal model. The PDSP is in accordance with disease progression and corresponds mainly to the PDMP. Moreover, both patterns overlap to a large extent with the human glucose metabolic PDRP. The multivariate PDSP expression scores are promising for preclinical evaluation of future therapeutic strategies that may modulate the observed patterns.

## Supplementary information

Supplementary material

## Data Availability

The datasets analysed during the current study are available from the corresponding author on reasonable request.
