# Supplementary material for: Identifying a glucose metabolic brain pattern in an adeno-associated viral vector based rat model for Parkinson’s disease using 18F-FDG PET imaging
Source: Sci Rep. 2019 Aug 26;9:12368. doi: 10.1038/s41598-019-48713-0 (PMC6710432; doi:10.1038/s41598-019-48713-0)
Supplement: Supplementary file 1 — Supplementary material [file 41598_2019_48713_MOESM1_ESM.docx]

**Identifying a glucose metabolic brain pattern in an adeno-associated viral vector based rat model for Parkinson’s disease using ^18^F-FDG PET imaging**

**Supplementary material**

Martijn Devrome^1^, Cindy Casteels^1^, Anke Van der Perren^2^, Koen Van Laere^1^, Veerle Baekelandt^2^, Michel Koole^1^

^1^Department of Nuclear Medicine and Molecular Imaging, KU Leuven, Leuven, Belgium

^2^Laboratory for Neurobiology and Gene Therapy, Department of Neurosciences, KU Leuven, Leuven, Belgium

**First Author:** Martijn Devrome, KU Leuven, Division of Nuclear Medicine, Herestraat 49, 3000 Leuven, Belgium, Telephone + 32 16 343715, Telefax +32 16 343759, E-mail: martijn.devrome@kuleuven.be (PhD candidate)

**Supplementary material**

Results of cylinder test at week 3, week 4, week 6 and week 9 for PD group and controls (HC). Data are expressed as percent use of the impaired forelimb relative to the total number of wall contacts.

|  | W3 | W4 | W6 | W9 |
| --- | --- | --- | --- | --- |
| PD1 | 0.45 | 0 | 0.05 | 0.05 |
| PD2 | 0.15 | 0 | 0.05 | 0.05 |
| PD3 | 0.3 | 0.1 | 0.05 | 0.05 |
| PD4 | 0.35 | 0.25 | 0.15 | 0.25 |
| PD5 | 0.3 | 0.1 | 0.05 | 0.05 |
| PD6 | 0.3 | 0.2 | 0.2 | 0.15 |
| PD7 | 0 | 0.05 | 0 | 0 |
| PD8 | 0 | 0 | 0 | 0 |
| PD9 | 0.55 | 0.3 | 0.2 | 0.15 |
| PD10 | 0 | 0 | 0 | 0.05 |
| HC1 | 0.45 | 0.45 | 0.3 | 0.3 |
| HC2 | 0.6 | 0.3 | 0.4 | 0.55 |
| HC3 | 0.55 | 0.6 | 0.35 | 0.3 |
| HC4 | 0.35 | 0.45 | 0.5 | 0.25 |
| HC5 | 0.65 | 0.35 | 0.45 | 0.5 |
| HC6 | 0.5 | 0.45 | 0.55 | 0.2 |
| HC7 | 0.8 | 0.5 | 0.6 | 0.55 |
| HC8 | 0.35 | 0.4 | 0.25 | 0.4 |
